# Achieving universal health coverage in Nigeria: the dilemma of accessing dental care in Enugu state, Nigeria, a mixed methods study

**DOI:** 10.1016/j.heliyon.2021.e05977

**Published:** 2021-01-22

**Authors:** Nkolika Uguru, Obinna Onwujekwe, Chibuzo C. Uguru, Udochukwu Ugochukwu Ogu

**Affiliations:** a Department of Preventive Dentistry, Faculty of Dentistry, College of Medicine University of Nigeria Enugu Campus, Nigeria; b Department of Health Administration and Management, Faculty of Health Sciences and Technology, University of Nigeria Enugu Campus, Nigeria; c Department of Oral and Maxillo-facial Surgery, Faculty of Dentistry, College of Medicine, University of Nigeria Enugu Campus, Nigeria; d Health Policy Research Group, Department of Pharmaco-therapeutics, College of Medicine, University of Nigeria, Enugu Campus, Nigeria

**Keywords:** Enugu, Nigeria, Universal health coverage, Dental care, Access to dental care

## Abstract

**Objective:**

Equitable access to oral healthcare is a major focus of the Universal health coverage debate in Nigeria. However, a great majority of the population still do not have full coverage for essential oral healthcare services. This study will determine the extent of inequities in accessing oral healthcare services and the factors influencing access to equitable oral healthcare in Enugu state Nigeria.

**Methods:**

A descriptive cross-sectional, urban and rural study conducted over two months in Enugu state Nigeria, using a mixed method approach. The quantitative study design used interviewer administered questionnaires to elicit information from 774 household members (394 urban and 380 rural) in study area who had sought dental care 6 months prior to study, and dental care providers (52) in selected dental health facilities. The qualitative study design involved in-depth interview of heads of selected dental health facilities to investigate factors influencing provision of dental care. Household data was collected house to house from randomly selected households in the LGA, while a face-to-face in-depth interview was conducted for purposively selected oral health professionals from study facilities.

**Results:**

Majority of respondents sought care when they had toothache (72%). There was inequity in utilization of dental care across socioeconomic status groups (SES). The least poor SES (Q5) sought dental care in the private facilities, and chose to have dental fillings more than the poorest (Q1) and very poor SES (Q2) who visited public facilities and patent medicine dealer shops more and opted more for tooth extractions.(p < 0.05) Cost of services influenced access and treatment choice more among Q1 and Q2 than Q5 (p < 0.05) Qualitative results show that facility location, low awareness, human resource shortage and oral health financing methods influenced access.

**Conclusion:**

Increased awareness and inclusion of oral healthcare in all health insurance schemes with expansion of current oral healthcare benefit package will improve access to care and further improve chances of attaining universal health coverage.

## 1 Introduction

Oral diseases affect half of the world's population with untreated dental caries affecting about 2.3 billion adults' worldwide [1], yet, oral health is still a neglected area of global health [2]. The unequal distribution of oral health personnel and the absence of appropriate facilities in many countries means disadvantaged communities have limited or no access to primary oral health care [3]. The importance of ensuring access to adequately trained oral health professionals and using a people-centered healthcare approach as a part of primary healthcare is critical to strengthen oral healthcare systems [4], and the universal health coverage mantra which proposes that all people have access to essential and quality healthcare without going through financial hardship to pay for it [5].

The Nigerian healthcare system has a three-tier structure, namely primary, secondary and tertiary. Oral health is also delivered at three levels with most oral health services, directed towards the provision of both rehabilitative and curative care [6]. Oral healthcare is also delivered in both public and private facilities however, these are inadequate and overstretched in many areas (Adeniyi et al). In most public facilities, oral health providers are co-located with other healthcare providers as these facilities are usually in-built in public hospitals and health centers, but the private oral healthcare facilities are usually stand-alone. Most advanced procedures are conducted in the teaching hospitals and basic procedures are usually done in primary healthcare facilities [7]. Public and private oral healthcare services are predominantly found in urban areas with a few mostly private oral health facilities found in rural areas [8]. There are more private oral health facilities than public in Nigeria, however the cost of dental treatment is less in public oral health facilities and as such, the public oral health facilities get more patronage [9].

Untreated dental caries in Nigeria at a prevalence of 90% is a huge problem [10, 11]. Similar estimates in Enugu state show prevalence of untreated dental caries at 97%, utilization of dental services at 15.5% in rural areas [12, 13] and 65.4% in urban areas [8]. This high prevalence of untreated caries with urban rural disparity in utilization, alludes to the inequity in access to oral healthcare services in the state. Enugu state, which is in the southeastern geopolitical zone of Nigeria, also operates the three tier health care system with a public private mix, however there are limited public facilities providing oral health care, with majority situated in the urban areas of the state [9]. The public facilities are funded from government budgetary allocations, while the private dental facilities are self-funded promoting exorbitant price setting [9].

Appropriate financing of oral healthcare is essential to ensure access to care. Financing for oral health care in the Nigerian public sector is derived from budgetary allocations to health at a very low rate of 5% of the national budget. Only a small proportion of total spending on health (0.41%) is allocated to oral health [6]. The private sector is largely independent of budgetary allocations and depend mainly on direct user fees. In a bid to ensure universal access to healthcare, equitable access to oral health services has been on the National agenda evidenced by the development of the National oral health policy in 2012. Despite the development of this policy, there is minimal impact as implementation rate is low, and a great majority of the population still do not have full coverage for essential oral healthcare services, neither is there any evidence of state level implementation of the policy [8]. Recently the Federal government intensified plans to review the 2012 policy and produce a 2020 national oral health policy, amidst all these, Enugu state like most states in Nigeria is yet to have a state oral health policy and as such implementation of the new national health policy might also prove challenging.

Dental insurance is the widely documented method of financial risk protection for oral health care and has had significant impact on dentistry and dental care use. A wide dental insurance coverage will reduce inequity and influence positively, people's decision to use dental care [14]. The majority of studies regarding demand for and utilization of oral health care services by insurance status have been conducted in developed countries. Such studies in Africa and many other developing countries are rare.

The benefit package for oral health in Nigeria's national health insurance plan is abysmal with very few basic benefits such as simple tooth extraction and amalgam filling. Consequently, citizens are faced with paying exorbitant out of pocket costs for most dental procedures. With this high treatment cost, access to quality dental care will prove difficult for people of low socioeconomic circumstances [15]). As minimal state level data exist, this study will measure the extent of inequities in accessing oral healthcare services and the factors associated with these inequities in Enugu state Nigeria. Findings from this study will contribute policy relevant evidence in development of subsequent oral health policies to improve access to care.

## 2 Materials and methods

### 2.1 Study area and setting

This article is part of a larger study conducted over two months in 2018 in Enugu State Nigeria. Enugu state is one of the 36 states of the Federal Republic of Nigeria, geographically, located in the southeast region of the country. It has a projected population of 4,411,100 million with urban population of about 1,032297, and rural population 2,235, 540 [16] Enugu state is made up of 17 Local Government Areas (LGAs); four urban and thirteen rural LGA's [16]. The Igbos are the predominant ethnic group found in the state, although people from other parts of the country reside in the state. The urban population is made up of mainly civil servants, traders, artisans and students/pupils of the various educational institutions in the state while the rural population is made up of government workers, artisans, subsistence farmers and traders. The study was conducted in two LGAs; one urban (Enugu East) with a projected population of 374,100 and the other rural LGA (Nsukka) with a projected population of 417,700) [17].

### 2.2 Study design and sampling

Both quantitative and qualitative research methods were employed concurrently, to conduct the study at the household and dental facility level. For the household study, a multi-stage sampling technique was used to select eligible households for the survey. Firstly, the LGAs were stratified into urban and rural areas. LGAs domiciling public dental facilities were then purposively selected. Secondly, a total of six political wards with population size ranging between 10,015 and 29,977 (see supplementary file for individual population sizes for wards) [17] (three in each LGA) were selected by simple random sampling. Households were selected by simple random sampling in each of the wards and household heads in the selected wards were interviewed to determine those that had ever experienced dental caries. Finally, households with members who had dental caries experience within six months from onset of study were selected and recruited into study until estimated sample size of 774 respondents was achieved. (394 in urban and 380 in rural) 116 households were visited in each of the rural wards while 126 households were visited in each of the urban wards giving each ward a representative sample based on total sample size. In every selected household, questionnaires were administered face-to-face to the head of household or representative and the household was the unit of analysis.

The facility-based study comprised 7 dental facilities (public and private) One public facility in the urban area and one in the rural area for adequate geographic representation; a major referral hospital for the two facilities in the state and four private dental clinics (2 each per local government area). The public dental facilities were purposively selected while the private facilities were selected by simple random sampling.

### 2.3 Qualitative data

An in-depth interview of purposively selected oral health professionals was done using a pretested interview guide. Fifteen oral health professionals of managerial cadre consisting of seven facility heads and eight directors and senior managers who were identified as being in the position to respond knowledgeably to the interview questions were selected. An audio taped face to face interview was conducted. Each interview lasted for about 35mins. The interviews were transcribed verbatim. The questions asked were designed to elicit information on the perspective of providers to challenges in accessing oral health care. Questions such as: How has your location affected how you provide dental services? How has your staff strength, influenced the type of services you provide? Are your patients always able to pay for the services? (See annex).

### 2.4 Data Analysis

Data Analysis was conducted with SPSS version 23 and STATA 12 software. Frequency and percentages were computed. Bi variate analysis was used to determine the test for associations and multivariate analysis was used to test factors that significantly influenced use of oral health services. All tests of significance were carried out at a p value of <0.05. Data was presented in tables and narratives in the result section.

SES index was used to categorize household respondents into SES quintiles: least poor, poor, most poor, very poor and poorest. Principal components analysis (PCA) was used to generate the SES index [18]. The input to the PCA was information on ownership of key assets together with the per capita cost of food. The SES index was disaggregated into quintiles with Q1 as the poorest and Q5 as least poor.

For the qualitative data, transcribed interviews were coded and analyzed using thematic content analysis. Themes were derived based on responses from participants on challenges to equitable access. Quotations and translations were checked and explanatory narratives were developed based on the quotes from respondents.

### 2.5 Ethical considerations

Ethical clearance was obtained from the University of Nigeria Teaching Hospital ethical review board prior to study. Verbal and written informed consent were obtained from respondents before interview commenced.

## 3 Results

Table 1 below shows 42.1% of the respondents were male, while 57.9% were female. 96.8% of respondents are educated and the SES quintile group was evenly distributed.

**Table 1:** Socio-economic/demographic characteristics of respondents.

Variables	Rural n (%) N = 380	Urban n (%) N = 394	Total n (%) N = 774
**Sex**			
Male	198 (25.6)	128 (16.5)	326 (42.1)
Female	182 (23.5)	266 (34.4)	448 (57.9)

**Age (Years)**			
Less than or equal 20	18 (2.3)	15 (1.9)	33 (4.3)
21 to 40	152 (19.6	250 (32.3)	402 (51.9)
41 to 60	167 (21.6	120 (15.5)	287 (37.1)
Above 60	43 (5.6)	9 (1.2)	52 (6.7)

**Marital status**			
Single	99 (12.8)	127 (16.4)	226 (29.2)
Married	266 (34.4)	238 (30.7)	504 (65.1)
Divorced/Widowed/Separated	15 (1.9)	29 (3.8)	44. (5.8)

**Religion**			
Christianity	378 (48.8)	392 (50.6)	770 (99.5)
Islam	2 (0.3)	2 (0.3)	4 (0.5)

**Educational status**			
Educated	358 (46.3)	391 (50.5)	749 (96.8)
Not Educated	22 (2.8)	3 (0.4)	25 (3.2)

**Highest level education**			
Primary	13 (1.7)	45 (6.0)	58 (7.7)
Junior	9 (1.2)	41 (5.6)	50 (6.8)
Senior	183 (24.4)	248 (33.0)	431 (57.4)
Tertiary	155 (20.6)	55 (7.5)	210 (28.1)

**Main Occupation**			
Unemployed	67 (5.0)	67 (8.7)	134 (17.3)
Subsistence farmer	39 (5.0)	13 (1.7)	52 (6.7)
Petty trader∗	58 (7.5)	188 (24.3)	246 (31.8)
Government worker	84 (10.9)	34 (4.4)	118 (15.2)
Private sector worker	30 (3.9)	21 (3.5)	57 (7.4)
Business man (SME)	90 (11.6)	44 (5.7)	134 (17.3)
Artisan	12 (1.6)	21 (2.7)	33 (4.3)

**Socio-Economic Status groups**			
Q1 (Poorest)	76 (20.1)	79 (20.1)	157 (20.3)
Q2 (Very poor)	75 (19.8)	79 (20.1)	153 (19.8)
Q3 (Most poor)	76 (20.1)	79 (20.1)	155 (20.0)
Q4 (Poor)	76 (20.1)	79 (20.1)	155 (20.0)
Q5 (Least poor)	76 (20.1)	78 (19.8)	154 (19.9)

∗ daily subsistence trader.

Table 2 below shows that geographic location, educational status, socioeconomic status of respondents, severe pain in tooth and oral health awareness, influence access to oral healthcare services (p < 0.05).

**Table 2:** Factors influencing access to oral health services.

Independent Variables	B	Std. Error	t	P value	Confidence Interval
(Constant)	1.534	2.712	0.566	.572	-3.791–6.858
Geographic location	.517	.081	6.410	.000∗	.358–.675
Age	-.002	.002	-1.137	.256	-.006–.002
Educational Status	.232	.174	.050	.000∗	.024–.054
Occupation	.001	.001	1.168	.243	-.001–.002
Socioeconomic status	.002	.001	2.120	.034∗	.014–.254
Cost (expenditure)	-.008	.000	.073	.942	.000–.000
Gender	.048	.062	.764	.445	-.075–.170
Marital status	.016	.045	.367	.714	- .071 - .104
Religion	.135	.393	1.334	.183	-.109–.573
Oral health awareness	.252	.090	.2.803	.004∗	.075–.428
No of people in household	.003	.019	.140	.889	-.035–.040
Knowledge of where to seek care	-.008	.017	-.462	.645	-.042–.026
Severe Pain in tooth	.275	.084	2.739	.002∗	.111–.439
Hole in teeth	2.119	.785	-2.700	.007	-3.659–-.578
Cheaper services	-.121	.146	-.827	.408	-.408–.166
Recommended by friends/family	.094	.110	.858	.391	-.122–.310
Qualification of staff	-.026	.025	-1.039	.299	-.075–0.23
Closeness to house	-.001	.013	-.099	.921	-.028–.025
Previous experience	-.054	.051	-1.046	.296.	-.154–.047
Staff attitude	.010	.099	.101	.920	-.184–.204

∗ Dependent Variable: utilization of dental services. R = 0.41.

Table 3 below shows that utilization of dental services had a serious financial impact on majority of the rural dwellers while urban dwellers recorded a minor financial impact. (p < 0.05). Out of pocket payment was the major source of oral healthcare financing across both locations. Health insurance was used minimally across both locations (p < 0.05).

**Table 3:** Payment coping mechanism.

Variables	Rural n (%) N = 380	Urban n (%) N = 394	Diff. X2 (P-value)	Total n (%) N = 774
Financial Effect of Dental Caries treatment				
No impact	29 (7.6)	5 (1.3)		34 (4.4)
Little impact	41 (10.8)	26 (6.6)		67 (8.7)
Minor impact	141 (37.1)	213 (54.1)		189 (24.4)
Serious impact	153 (40.3)	114 (28.9)		354 (45.7)
Very serious impact	16 (4.2)	36 (9.1)		130 (16.8)
**Total**	380 (100)	394 (100)	1.811 (0.01)	774 (100)

Sources of fund for Treatment				
Cut down spending	13 (3.4)	68 (17.3)		81 (10.5)
OOP	359 (94.5)	296 (75.1)		655 (84.6)
Health insurance	5 (1.3)	10 (2.5)		15 (1.9)
Cash donations	3 (0.8)	20 (5.1)		23 (3.0)
**Total**	380 (100)	394 (100)	74.993 (0.01)	774 (100)

Table 4 below shows majority of the people do more dental extractions than dental fillings irrespective of SES. However, respondents in poorest group have more extractions than those in least poor group. Respondents in the least poor SES groups (Q5) fill their teeth more than those in the poorest SES groups (p < 0.05). The poorer SES groups (visit Public dental facilities and patent medicine dealers more than the least poor SES. (p < 0.05). More respondents across all the SES groups felt serious financial impact of visiting the dentist. However, the poorest SES felt it more. The least poor SES, feel minor impact of the disease more than the poor SES. Out of pocket is the main payment mechanism for accessing dental services across all SES groups however the poorest (Q1) group depend on cash donations and borrowed funds more than the least poor (Q5) SES.

**Table 4:** Inequities in access to dental care across socio-economic status groups.

Variables	Q1 n (%) Poorest N = 157	Q2 n (%) Most Poor N = 153	Q3 n (%) Poorer N = 155	Q4 n (%) Poor N = 155	Q5 n (%) least poor N = 154	Chi square	P value
Type of treatment Done							
Dental filling	26 (16.6)	29 (19.0)	44 (28.4)	38 (24.5)	46 (29.9)		
Extraction	131 (83.4)	124 (81.0)	111 (71.6)	117 (75.5)	108 (70.1)		
**Total**	**157 (100)**	**153(100)**	**155 (100)**	**155 (100)**	**154 (100)**	11.531	0.02∗

Where treatment was sought							
Private dental facility	21 (13.5)	49 (32.0)	78 (50.3)	68 (43.9)	69 (44.8)		
Public dental facility	101 (64.7)	67 (43.8)	44 (28.4)	54 (34.8)	40 (26.0)		
Itinerant drug peddler	4 (1.9)	7 (5.4)	14 (9.0)	16 (10.3)	30 (19.5)		
Patent medicine dealer/pharmacy	31 (19.9)	30 (19.6)	19 (12.3)	17 (11.0)	15 (9.7)		
**Total**	**157 (100)**	**153(100)**	**155 (100)**	**155 (100)**	**154 (100)**	11.329	0.01∗

Factors influencing treatment choice							
Cost of treatment	28 (18.9)	37 (24.2)	23 (14.8)	31 (20.0)	30 (19.5)		
Severity of disease	23 (14.7)	27 (17.6)	41 (26.5)	39 (25.2)	41 (26.6)		
Recommendation from friends/family	91 (58.3)	79 (51.6)	76 (49.0)	67 (43.2)	65 (42.2)		
Previous experience	15 (8.1)	10 (6.6)	15 (9.7)	18 (11.6)	18 (11.7)		
**Total**	**157 (100)**	**153(100)**	**155 (100)**	**155 (100)**	**154 (100)**	22.730	0.27

Financial Impact of treatment							
No impact	6 (3.8)	4 (2.6)	4 (2.6)	7 (4.5)	13 (8.4)		
Little impact	9 (5.7)	16 (10.5)	20 (12.9)	10 (6.5)	12 (7.8)		
Minor impact	22 (14.0)	28 (18.3)	46 (29.7)	50 (32.3)	43 (27.9)		
Serious impact	76 (48.4)	70 (45.8)	66 (42.6)	73 (47.1)	69 (44.8)		
Very serious impact	44 (28.0)	35 (22.9)	19 (12.3)	15 (9.7)	17 (11.0)		
**Total**	**157 (100)**	**153(100)**	**155 (100)**	**155 (100)**	**154 (100)**	55.668	0.01∗

Payment mechanism							
Cut down other expenses	30 (19.1)	13 (8.5)	14 (9.0)	15 (9.7)	9 (5.8)		
Out of pocket payment	117 (74.5)	131 (85.6)	135 (87.1)	136 (87.7)	136 (88.3)		
Cash donations	10 (6.4)	9 (5.9)	11 (3.9)	4 (2.6)	9 (5.9)		
**Total**	**157 (100)**	**153(100)**	**155 (100)**	**155 (100)**	**154 (100)**	30.378	0.04∗

∗p < 0.05.

In-depth interview findings shown below explored additional perspective of providers on factors that affect equitable access to oral health services.

### 3.1 Geographic location

Some respondents emphasized the importance of geographic location, citing the low patient turnout in their facility to distance from the city and long patient travel time. More public facilities are needed in the LGAs to increase access to care.

*“We don't get a large number of patients because the facility is far from town. When you add the transport cost with treatment cost and inconvenience of travelling to the teaching hospital, a lot of patients opt out for clinics in town. (Rural/Public)*

### 3.2 Human resource number and need

Human resource shortage both in number and staff mix reduce access to appropriate oral healthcare. The rural public facility is the hardest hit by this human resource challenge whereby the dentist who is the only staff is unable to function maximally. However the urban facilities have better staffing.

*“I'm all alone here. If I need a second opinion I don't have anybody to turn to. So that is another challenge I'm facing in providing service.” (Rural/Public)…. We have enough doctors and dental staff to meet the needs of our patients. We have the full complement. We have enough staff, at least to meet our needs (Urban/Public)*

### 3.3 Cost and payment coping mechanisms

Majority of the population pay out of pocket and so find dental treatment cost high. They usually treat at home or visit cheaper drug shops. *“My patients complain of high cost (Urban/Private)…some of them cannot afford to see the dentist so they go to drug peddlers and when it get really bad they now come to my clinic and we can only extract which is the cheapest treatment (Rural/private*).

Majority of respondents said dental health insurance was unavailable for majority of the patients as only a fraction of the population are covered by health insurance. Dental insurance has not been properly promoted under the NHIS leading to low involvement by dentists. Quotes from respondents have been given below:

*“NHIS is not serious about dentistry. The benefit package is really poor. The amount of money they put for a procedure is so low that a lot of dentists don't like taking insurance patients”. (Urban/Public)*

*“Well to be frank with us, NHIS has not really tried in the dental aspect. The most covered treatment choice is extraction. If a patient opts out of an extraction, then any other treatment becomes too expensive. NHIS don't cover it and so that means they are encouraging patients to remove their teeth. So, treatment option for anybody that is under NHIS is either scaling and polishing or extraction (Urban/private). There are other insurance types, mainly private health insurance which is better but not available to everyone (Urban/private).*

### 3.4 Government policies and taxation

Respondents opined that government policy makers do not realise the importance of dental health or its link to general health and so under-represent it in key healthcare decisions and policies in the country. *“These government people don't know anything about dentistry, so much importance is not attributed to it (Urban/public)...…..”Yes. At the policy level yes. In fact, at the policy level it is very important to make things better. Now so many people are going for the NHIS medically because they are seeing the benefit but most of them are not going in dental because they don't see any benefit. It's either I wash my teeth or I remove it.” (Urban/private)*

*“There is nothing like fee exemption or subsidy and I don't think there is anything in the government policy that says that. If there is, I have not seen (Rural/private)” We just give discount for some people period. Maybe the government hospital can give full waiver but how can private do that? We are struggling too in this harsh economy (Urban/private).*

*In a public center, you do not have the power to give any waiver or subsidy. If you must, permission must be sought and the go-ahead given by management before that can be done ‘(Urban/public)*

### 3.5 Low oral health awareness

Respondents opined that most members of the population do not know enough about dental care. Most see it only as a place to have their teeth removed when they have a toothache. *I just believe most of the problem we have is poor awareness (Rural/Private*).

## 4 Discussion

Our study shows that access to oral healthcare is mainly influenced by geographic location, educational status, socioeconomic status, oral health awareness and toothache. We observed that as level of education increases, individuals access oral healthcare more. Increased level of education could also be synonymous with increased oral health awareness which also improves access to care. Contrarily our study shows that majority of our respondents avoid regular dental checks and mainly access care when they are in pain. This could be attributed to the high cost of dental care. This finding is similar to other studies which show that dental caries is a very expensive disease to treat and most patients visit the dentist only when they are in pain [18].

Level of awareness has also been implicated as a factor influencing access to dental care. Our study results showed that increased oral health awareness increased access to care. Also low oral health awareness especially in the rural areas (who need more outreaches, especially in the remote areas) has been linked to poor utilization of care or late visit to the dentist necessitating mainly extractions. Other studies have also shown that low utilization of dental care is a function of low awareness as some sociodemographic factors such as level of education are known to influence awareness about available oral health-care services and thus, could either promote or mitigate access to care [19, 20, 21].

Our study observed that accessing dental care had a serious financial impact on majority of the respondents in the rural areas and minor financial impact on majority of urban dwellers. We also observed that the poorest SES felt the financial impact more other SES groups. These could be because the poverty index of the rural area is low. A study [22] found that the poverty level of Nsukka (the rural area of our study) is 69.2%, with the level of socioeconomic development being low with a composite availability index of 580. Also, it could be a consequence of the limited number of public oral health facilities in the rural areas. Asides from the limited number of public oral health facilities, our study shows inadequate dental staff at the public facility thus the facility is unable to meet the oral health needs of the rural population. This will definitely sway patients to the more expensive private dental facilities in that area or even make them resort to home treatment or use of itinerant drug peddlers who often complicate illness, thus bringing said patient back to the expensive private dental facility. These private facilities are now able to set prices which may be out of reach of most low-income rural dwellers. This is the reverse in the urban areas where there is a larger spread of public oral health facilities. This lopsided distribution of oral health facilities with more facilities in urban than rural alludes to an inequity in access to care based on location. This is because in Enugu state like the rest of Nigeria, majority of the population live in the rural areas and with low number of facilities, equitable access to quality oral healthcare will be further compromised. This inequitable distribution of oral health facilities in Nigeria has been corroborated by other studies [6].

Universal health coverage ensures that all people receive affordable quality healthcare irrespective of their financial status. Dental insurance reduces the burden of paying for oral healthcare and improves access and utilization of dental care services [3, 23], but both consumers and providers seem ignorant of financial risk protection mechanisms available to reduce the burden of payment for dental treatment. In Nigeria, dental health insurance is grossly underused. This scenario is similar in many low and middle-income countries [24]. In developed countries, a similar scenario is played out amongst refugees and ethnic minority groups [25]. In Nigeria, this problem may emanate directly from the design of the country's social health insurance scheme which has a very limited benefit package for dental care, which consists of only dental checkup, scaling and polishing, simple tooth extractions, amalgam fillings and maximum of four dentures [26].Most health management organizations do not include any dental package in their insurance plan. Those who do, have a very limited package with poor coverage and most dental healthcare providers are not very conversant with the workings of the dental health insurance plan. Thus, many do not register with any plan, depriving consumers the option of using an insurance plan. Another factor that might promote inequity in access to oral care is the fact that oral health is categorized as secondary care in the National health insurance plan. This will inadvertently put dental care out of reach of the poor and vulnerable, exposing them to cheaper treatment at non-qualified personnel. This finding is synonymous with the report from Ifijeh [27].

In conclusion, achieving universal health coverage without taking into consideration equitable access to oral healthcare is not feasible. In addition, this study observed that inequity in access to oral healthcare in Enugu state is largely due to lopsided distribution of oral health facilities to favour urban areas, low awareness of oral health by policy makers and citizens, inefficient health financing mechanism for oral health. In order to achieve a key mandate of universal health coverage which is equitable access to good quality healthcare, the financial mechanism and proper distribution of facilities and staff need to be taken into consideration. The restructuring of the benefit package to expand services covered and categorization of oral healthcare into primary care in the national health insurance scheme will equally increase access to care.

## Clinical significance

Dental treatment in a resource poor environment like Nigeria is mostly out-of-pocket. Without dental health insurance, most individuals would be unable to afford much needed dental care and thus postpone seeking care. Patients that would otherwise benefit from advanced restorative treatment would have no option but to extract their teeth.

## Declarations

### Author contribution statement

N. Uguru: Conceived and designed the experiments; Performed the experiments; Analyzed and interpreted the data; Contributed reagents, materials, analysis tools or data; Wrote the paper.

O. Onwujekwe: Conceived and designed the experiments; Contributed reagents, materials, analysis tools or data; Wrote the paper.

U. Ogu: Performed the experiments; Analyzed and interpreted the data; Contributed reagents, materials, analysis tools or data; Wrote the paper.

C. Uguru: Analyzed and interpreted the data; Contributed reagents, materials, analysis tools or data; Wrote the paper.

### Funding statement

This research did not receive any specific grant from funding agencies in the public, commercial, or not-for-profit sectors.

### Data availability statement

Data will be made available on request.

### Declaration of interests statement

The authors declare no conflict of interest.

### Additional information

No additional information is available for this paper.

## Appendix A Supplementary data

Enugu State New Ward Population.xls Enugu State New Ward Population.xls
